# MATERIALS SCIENCE: Bringing Biolubricants to Industry

**DOI:** 10.1289/ehp.117-a488a

**Published:** 2009-11

**Authors:** Carol Potera

**Affiliations:** **Carol Potera**, based in Montana, has written for *EHP* since 1996. She also writes for *Microbe, Genetic Engineering News*, and the *American Journal of Nursing*

Up to 50% of lubricants used around the world are lost to the environment through evaporation, spills, leaks, and improper disposal, and contaminate soil and groundwater. “It’s an underappreciated source of pollution,” says Brajendra K. Sharma, a visiting research chemist at the U.S. Department of Agriculture (USDA) National Center for Agricultural Utilization Research in Peoria, Illinois. Now vegetable-based biolubricants offer an alternative to petroleum-based lubricants in a range of machinery, from elevators to ski lifts to chainsaws.

Vegetable oils have inherent disadvantages that limit their use in industrial applications, mainly destruction by oxygen, deterioration at high temperatures, and solidifying at low temperatures. Sharma and his colleagues designed a process to overcome these problems that requires a few simple steps such as adding an antioxidant. According to the researchers, the process generates no toxic products and uses existing equipment at facilities that manufacture petroleum-based lubricants.

“Simple chemistry and compatibility with existing operations are keys to getting industry to adopt our lubricants,” says Sevim Erhan, formerly a chemist at the Peoria center and now director of the USDA Eastern Regional Research Center in Wyndmoor, Pennsylvania. She adds, “Our main goal is to make 100% biodegradable lubricants with nonpolluting processes to replace petroleum products.” One antioxidant that has proven especially effective, zinc dialkyldithiocarbamate, is “not considered hazardous,” according to its Material Safety Data Sheet but nonetheless should be kept out of contact with soil, waterways, drains, and sewers.

Because performance requirements of lubricants differ for various industries, the team is exploring additional chemical modifications. In the September 2009 issue of the *Journal of Agricultural and Food Chemistry*, for instance, Sharma and colleagues described how they converted oleic acid to a stable form by adding formic acid and hydrogen peroxide, using aniline as a catalyst, and heating the mixture for several hours. This “one pot” synthesis was easily performed and gave good yields, and the liquids used were recovered and recycled.

A few industries have adopted biolubricants. Since 2002, elevators in the Statue of Liberty have run on biodegradable hydraulic fluid. Hydraulic systems are prone to leaks, leaving puddles at the bottom of elevator shafts that run deep underground, raising the risk of ground water contamination. “But soybean oil readily degrades, and there’s little harm to the environment,” says Jack Stover, president of Agri-Lube, Inc., in Defiance, Ohio, who licensed the USDA technology. The soybean-based hydraulic fluid shows excellent anti-friction and anti-wear properties and is less flammable than petroleum-based counterparts.

Penn State University in University Park switched all campus elevators and farm equipment to biodegradable hydraulic fluid. “About 35,000 gallons of oil in elevators has been replaced, and that’s a significant amount,” says Joseph Perez, a senior research scientist in the university’s Department of Chemical Engineering.

Aluminum producer Alcoa, Inc., changed to biolubricants for its flat-rolling operations. During rolling, thick slabs of aluminum are flattened under heavy rollers into thin sheets that are shaped into vehicle panels, doors, beverage cans, and more. Lubricants are used in such operations to hold the aluminum and rollers together and dissipate heat. Ronald Reich, a technical consultant at Alcoa, collaborated with the USDA to create a biolubricant to replace petroleum-based lubricants that emit volatile organic compounds, which can cause eye and airway irritation, headaches, and other health problems. Because biolubricants are less volatile than petroleum-based lubricants, “we use less biolubricant to do the same job at a cost savings,” says Reich.

Generally, biolubricants cost about twice as much as petroleum-based products, which hinders their adoption by more industries. “Biodegradable lubricants are a niche market right now,” says Perez. However, a rise in crude oil prices could make biolubricants more cost-competitive. “Ultimately, it all boils down to cost,” says Sharma.

